# Non-Dominant Hemisphere Excitability Is Unaffected during and after Transcranial Direct Current Stimulation of the Dominant Hemisphere

**DOI:** 10.3390/brainsci14070694

**Published:** 2024-07-12

**Authors:** Erik W. Wilkins, Richard J. Young, Daniel Houston, Eric Kawana, Edgar Lopez Mora, Meghana S. Sunkara, Zachary A. Riley, Brach Poston

**Affiliations:** 1 Department of Kinesiology and Nutrition Sciences, University of Nevada, Las Vegas, NV 89154, USA; wilkie1@unlv.nevada.edu; 2 Interdisciplinary Ph.D. Program in Neuroscience, University of Nevada, Las Vegas, NV 89154, USA; ryoung@unlv.edu; 3 School of Medicine, University of Nevada, Las Vegas, NV 89154, USA; houstond@unlv.nevada.edu (D.H.); kawana@unlv.nevada.edu (E.K.); lopeze54@unlv.nevada.edu (E.L.M.); 4 Department of Biology, Virginia Commonwealth University, Richmond, VA 23284, USA; sunkaram@vcu.edu; 5 Department of Kinesiology, Indiana University Purdue University Indianapolis, Indianapolis, IN 46202, USA; zariley@iupui.edu

**Keywords:** transcranial direct current stimulation, transcranial magnetic stimulation, cortical excitability, motor evoked potential, motor skill

## Abstract

Transcranial direct current stimulation (tDCS) increases primary motor cortex (M1) excitability and improves motor performance when applied unilaterally to the dominant hemisphere. However, the influence of tDCS on contralateral M1 excitability both during and after application has not been quantified. The purpose was to determine the influence of tDCS applied to the dominant M1 on the excitability of the contralateral non-dominant M1. This study employed a double-blind, randomized, SHAM-controlled, within-subject crossover experimental design. Eighteen young adults performed two experimental sessions (tDCS, SHAM) in counterbalanced order separated by a one-week washout. Transcranial magnetic stimulation (TMS) was used to quantify the excitability of the contralateral M1 to which anodal tDCS was applied for 20 min with a current strength of 1 mA. Motor evoked potential (MEP) amplitudes were assessed in 5 TMS test blocks (Pre, D5, D10, D15, and Post). The Pre and Post TMS test blocks were performed immediately before and after tDCS application, whereas the TMS test blocks performed during tDCS were completed at the 5, 10, and 15 min stimulation timepoints. MEPs were analyzed with a 2 *condition* (tDCS, SHAM) × 5 *test* (Pre, D5, D10, D15, Post) within-subject ANOVA. The main effect for *condition* (*p* = 0.213), the main effect for *test* (*p* = 0.502), and the *condition* × *test* interaction (*p* = 0.860) were all not statistically significant. These results indicate that tDCS does not modulate contralateral M1 excitability during or immediately after application, at least under the current set of common tDCS parameters of stimulation.

## 1. Introduction

Transcranial direct current stimulation (tDCS) is a non-invasive brain stimulation method that involves the application of a low-level electrical field to a specific brain area to modulate cortical excitability and influence motor performance [1,2,3,4,5,6,7,8,9]. The effects of tDCS are largely determined by the orientation of the electrical field generated by the positions and polarity of the electrode montage [2,3]. The most widely studied electrode montage involves an electrode pair consisting of an anode and a cathode, which results in current flowing from the cathode to the anode and, usually, an increase in excitability of the targeted brain area underlying the anode (termed anodal tDCS) [1,6,7,8]. For example, placement of the anode over the primary motor cortex (M1) and the cathode over the contralateral supraorbital (SO) region (M1-SO electrode montage) usually leads to increased M1 excitability after stimulation ends as measured by increases in motor evoked potential (MEP) amplitude elicited by transcranial magnetic stimulation (TMS) [7,8,10,11]. In addition, other bihemispheric and dual-source electrode montages involving anodal tDCS of the left M1 have also demonstrated similar effects on M1 excitability [12,13,14,15].

The vast majority of motor system tDCS studies have utilized the SO-M1 electrode montage and anodal tDCS to target the dominant left M1 that primarily controls the contralateral dominant right hand. Furthermore, a minority of studies have involved the opposite arrangement of anodal tDCS applied to the non-dominant right M1 to impact the motor performance of the contralateral non-dominant left hand [1,6,7,9,16,17]. In both cases, most of the literature has reported significant M1 excitability increases of about 20–40% and motor skill improvements of about 10–15% after a single tDCS application of 10–20 min with current strengths of 1–2 mA [1,6,7,9,16,18,19,20,21]. However, it appears that approximately 20–30% of studies have reported no significant effects on these same outcome measures [1,6,22]. In addition, the motor skill enhancements observed in these studies have been attained when tDCS is applied both before and during motor task practice. Although it is generally thought that tDCS applied during motor task practice is more effective, few studies have directly and systematically compared stimulation timing in the same study using the same participants. Nonetheless, most of the tDCS studies that have observed the greatest increases in motor learning [21,23,24] have targeted the dominant left M1 using the SO-M1 electrode montage concurrent with motor practice using the aforementioned stimulation parameters.

Despite the large amount of research on left dominant M1 excitability and the motor performance of the contralateral right dominant hand that it primarily controls using the SO-M1 montage, relatively little is known about the effects of this paradigm on the unstimulated right non-dominant M1 and corresponding left non-dominant hand. This is especially the case in the time period during the application of tDCS to the left M1, where it appears that no studies have undertaken concurrent TMS measurements from the right M1. On the other hand, a small number of studies have conducted TMS or brain imaging assessments of right M1 net excitability, activity, or transcallosal effects after delivery of tDCS to left M1. For example, in an early small-scale study, Lang et al. (2004) [25] reported that left M1 tDCS had no effects on right M1 excitability as indicated by MEP amplitudes collected from the left hand immediately and 40 min after stimulation. More recently, an extensive study used magnetic resonance spectroscopy (MRS) to measure gamma-aminobutyric acid (GABA) levels in both M1s following tDCS of the left M1. The results revealed a decrease in GABA in both the stimulated left M1 and the unstimulated right M1 during and after tDCS application. These results implied that tDCS of the left M1 led to less inhibition and greater excitation in both the left and right M1s [14]. However, TMS measurements were not taken in the study to confirm an overall increase in net excitation of the right M1. In another study, anodal tDCS did not influence net contralateral M1 excitability, but did increase the amount of interhemispheric inhibition (IHI) as assessed by paired-pulse TMS exerted onto the contralateral M1, which is mediated by transcallosal pathways [26]. However, these results were obtained by right M1 anodal tDCS application and the effects were measured in the left M1, which may not give the same results expected by the opposite arrangement of tDCS of left M1 effects on right M1. This is because the amount of IHI from the dominant hemisphere to the non-dominant hemisphere is greater than vice versa [27]. In addition, separate previously identified transcallosal pathways that mediate interhemispheric facilitation (IHF) were also not measured in the study. In regard to motor performance, only a few studies have investigated the influence tDCS of the left M1 using the SO-M1 montage on the motor performance of the ipsilateral left hand that is primarily controlled by the right M1. These studies have yielded mixed results as two found a very small non-statistically significant increase in left hand motor performance [28,29], and one found a trend of a decrease [30]. Thus, the effects tDCS of left M1 using the SO-M1 montage on right M1 excitability and motor performance of the corresponding left hand remain unclear even in healthy adults, especially during stimulation.

The lack of direct research on the aforementioned topics is surprising given the promising results of tDCS of the left M1 for important practical applications such as motor learning, motor rehabilitation, and performance of vital activities of daily living [1]. For example, the viability of tDCS as a long-term intervention with wide-ranging real-world applications would be questionable if improvements elicited in the right hand would be accompanied by left hand performance being simultaneously degraded or unchanged. Furthermore, while the basic view that upper limb muscles are primarily controlled by the contralateral M1 is well supported, this does not mean that the ipsilateral (right) M1 has little to no effects on unimanual voluntary movements of the ipsilateral (right) hand that is primarily controlled by the left M1 [31,32]. In fact, accumulating evidence supports the importance of the ipsilateral (right) M1 for certain aspects of not only voluntary movement production such as movement initiation [33,34,35], but also motor learning processes in the ipsilateral (right) hand [13,36,37,38,39]. Thus, it is imperative to ultimately understand the effects tDCS of the left M1 using the M1-SO montage on the unstimulated non-dominant contralateral right M1 in order to elucidate the effects of tDCS on motor skill acquisition of unilateral movements performed by each of the hands. However, a logical first step would be to investigate changes in right M1 excitability in resting conditions without the complicating factors of simultaneous or previous background muscle activation due to muscle contraction in general or specific motor practice. 

The purpose was to determine the influence of tDCS applied to the dominant left M1 on the excitability of the unstimulated contralateral non-dominant right M1. In particular, the primary interest was to characterize any changes in right M1 excitability during tDCS of the left M1 using the typical SO-M1 electrode montage, whereas right M1 excitability immediately after tDCS was of secondary interest. This was accomplished by measuring MEP amplitudes in the left first dorsal interosseous (FDI) muscle at rest in response to TMS applied to the right M1 in test blocks performed before, during, and after anodal tDCS was delivered to the left M1 for 20 min. Based on a prior MRS study [14], it was hypothesized that MEP amplitudes obtained from the right M1 would be greater both during and immediately after tDCS of the left M1 compared to the SHAM condition. Thus, it was also hypothesized that MEP amplitudes would be increased relative to the baseline at timepoints during and immediately after stimulation in the tDCS condition, but not in the SHAM condition.

## 2. Materials and Methods

### 2.1. Participants

A total of 18 individuals took part in this study (11 males and 7 females; average ± standard deviation age: 24.1 ± 4.0 years). Written informed consent was obtained from all volunteers prior to participation. All participants were strongly right-handed, as confirmed by the Edinburgh Handedness Inventory [40]. Participants were screened to ensure they met international non-invasive brain stimulation criteria [41]. Additional inclusion criteria included (1) the ability to provide informed consent; (2) being free from any known neurological or psychiatric condition; and (3) being 18–45 years of age. Exclusion criteria included (1) an uncontrolled medical condition; (2) metal in the skull or eye such as a cardiac pacemaker, brain stimulator, shrapnel, surgical metal, clips in the brain, cochlear implants, and metal fragments in the eye; (3) diagnosed hearing loss; (4) having had a brain tumor, a stroke, head trauma, epilepsy, or a history of seizures, having a neurological disorder or movement disorder, or having a head injury that involved being passed out for more than a few seconds; and (5) being pregnant or thought to be pregnant. The study adhered to the principles of the Declaration of Helsinki and received approval from the Biomedical Institutional Review Board at the University of Nevada, Las Vegas. 

### 2.2. Experimental Design and Protocol

This study employed a double-blind, SHAM-controlled, randomized, within-subject experimental design. The within-subject design was chosen to mitigate the substantial inter-individual differences in the responsiveness to tDCS that are thought to be due to a combination of a wide range of anatomic, biological, physiological, and genetic factors [42,43]. In addition, within-subject designs allow for more statistical power compared with between-subject designs [44]. The order of presentation of the tDCS and SHAM conditions was assigned to participants using an online application (Research Randomizer; www.randomizer.org) by a member of the research team who did not participate in the collection of data [45,46]. All participants underwent two experimental sessions that were conducted at the same time of day and spaced 7 days apart [47,48], which is the most common washout period used in tDCS studies. Both sessions were identical except for the type of stimulation applied (tDCS, SHAM). 

**Figure 1:**
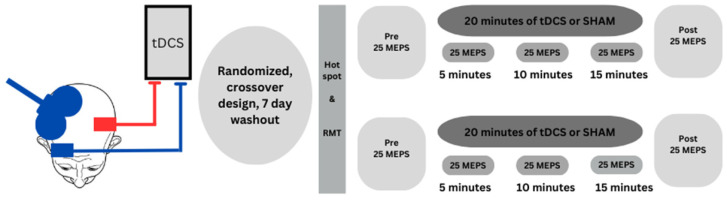
Schematic illustration of the study design and major experimental procedures. tDCS SO-M1 electrode montage placement over the left M1 and TMS coil placement over the right M1 FDI motor hotspot. tDCS or SHAM stimulation was delivered to left M1 for 20 min using the standard M1-SO electrode montage (anode over the left M1, cathode over the right eyebrow) while MEPs were evoked from the right M1 and collected from the corresponding left FDI in a total of five TMS test blocks (Pre, D5, D10, D15, Post) performed before (Pre), during (D5, D10, and D15 min timepoints), and immediately after (Post) stimulation.

Each session lasted approximately 1.25 h and followed a specific sequence of steps: (1) TMS was applied to the scalp over the left M1 to determine the motor hotspot location of the FDI muscle of the right hand. This was followed by determination of the resting motor threshold (RMT) and the 1 mV MEP stimulation intensity (SI) as a percentage of maximum stimulator output (%MSO) for the right FDI; (2) the tDCS electrode montage was placed over the left M1 FDI motor hotspot location and held in place by a tightly fitting scalp cap. However, the stimulator was not turned on at this time. (3) TMS was applied to the scalp cap over the right M1 to determine the motor hotspot location of the FDI muscle of the left hand. Subsequently, RMT and the 1 mV MEP SI was determined for the left FDI. (4) The Pre TMS test block was performed and MEPs were evoked from the right M1 and collected from the corresponding left FDI; (5) 20 min of tDCS or SHAM stimulation was applied to the left M1 while TMS test block was undertaken. Thus, MEPs were evoked from the right M1 and collected from the corresponding left FDI concurrent with the ongoing 20 min of stimulation. Data collection for these TMS test blocks started at the 5, 10, 15 min timepoints during stimulation (termed D5, D10, and D15); and (6) the Post TMS test block was performed and MEPs were evoked from the right M1 and collected from the corresponding left FDI. Therefore, MEPs attained during all the TMS test blocks were always evoked from the right M1 and collected from the corresponding left FDI. Figure 1 illustrates these major experimental steps, with detailed methodological descriptions provided in subsequent sections. Throughout all experimental conditions, the investigators conducting the experiments remained blinded to the stimulation condition applied to participants. Accordingly, the investigator who operated the tDCS device and administered stimulation was not involved in any other experimental procedures [45,46].

### 2.3. Experimental Procedures

#### 2.3.1. TMS Measurements and EMG Recording

TMS was administered utilizing a Magstim 2002 device coupled to a double 70 mm figure-of-eight remote-controlled coil. The coil was placed in contact with the surface of the scalp and orientated tangentially at an angle of 45 degrees to the sagittal plane with the handle pointed laterally and backwards. This typical arrangement induces a posterior to anterior directed current flow in M1 to elicit MEPs in the contralateral hand. Accordingly, MEPs were evoked in the right and left FDIs using single-pulse TMS of the left and right M1s, respectively. All MEPs were induced while the FDI muscle of either the left or right hand was maintained in a state of complete rest. 

The electromyographic (EMG) activity of the FDI muscle of each hand was measured using two surface electrodes that were arranged in a belly tendon montage while a ground electrode was placed on the back of the hand. The EMG signals were acquired and recorded employing hardware (1902 amplifiers, micro 1401 data acquisition interface) and software (Signal 5.04) provided by Cambridge Electronic Design (Cambridge, UK). The posture of the participants and experimental arrangement were similar to prior studies when MEPs were elicited from the FDI of the right hand [21,49]. Analogously, the same set of general experimental conditions were used when MEPs were collected from the left hand in the present study. Briefly, participants were seated upright in a chair with their hand resting palm down on the surface of a small table beside them. The forearm was also resting on the table surface with the wrist in neutral, the elbow flexed to an angle of ~90 degrees, and the shoulder abducted to ~45 degrees. Participants were directed to keep their eyes open and maintain this posture for all TMS measurements as it has been clearly shown that MEP amplitude in hand muscles can vary with changes in shoulder and upper limb position [50,51]. Importantly, participants were provided with visual feedback of their FDI EMG activity during all TMS testing to make certain that the FDI remained at rest at all times. FDI EMG was displayed online in real time on a computer monitor located ~0.75 m in front of the participants [52]. Detailed instructions were given to the participants on how to use the visual feedback to keep the FDI at rest at all times. Finally, one investigator continually monitored the body posture and FDI EMG level of the participants to ensure that they constantly adhered to these requirements.

To determine the motor hotspot of the FDIs, TMS pulses were given to the presumed area over the scalp corresponding to the hand representation area until the point that evoked the largest MEP in the resting FDI was identified [53]. This point was denoted as the stimulation site for each M1 and marked with a temporary marker for all subsequent RMT, 1 mV SI, and MEP measurements in the TMS test blocks. The point was marked directly on the scalp for left M1 testing and on tape placed on a scalp cap for right M1 testing (see below). RMT was measured in each FDI at rest using common methodology and was defined as the lowest TMS SI as a %MSO that could produce an MEP with an amplitude greater than 50 microvolts in at least 5 out of 10 consecutive trials. RMT was quantified in the FDI of both hands mainly because some studies [54,55] have indicated that individuals with lower RMT values may display a larger increase in MEPs following tDCS application. Therefore, if any increases in MEP amplitude were observed due to tDCS, these values could be correlated with the RMT for each participant. In addition, RMT served as a basic baseline and control measure of cortical excitability for each FDI on each of the two experimental days. For the 1 mV MEP SI of the left and right M1s, a great amount of care was taken to identify the TMS SI (%MSO) that would elicit an average MEP of as close to 1 mV as possible for a TMS block of 25 trials. Twenty-five MEP trials per block was chosen as this number has been shown to represent the best trade-off between experimental time efficiency and obtaining valid MEP amplitude averages for blocks of TMS trials [56]. The 1 mV SI determination was carried out utilizing methodology from two previous studies [21,49]. Briefly, TMS pulses were initially delivered at ~55 %MSO and this SI was adjusted while MEPs were monitored and quantified online by the Signal software. Once it appeared that MEP amplitudes were as close as possible on average to 1 mV, the Signal software program was reset and the Pre TMS test block was collected. This assured that the Pre TMS test block MEP amplitude was very close to 1 mV in both stimulation conditions performed on the two days. Moreover, this avoided any potential confounds due to presence of substantially different baseline MEP amplitudes before tDCS was applied in the two stimulation conditions. Subsequently, the same 1 mV SI as a %MSO was used to evoke all MEPs in the following D5, D10, D15, and Post blocks of TMS testing for each participant on a given day. Finally, the inter-trial interval (ITI) between consecutive MEPs was set to 6 s for all TMS test blocks.

#### 2.3.2. tDCS Application

A NeuroConn DC Stimulator Plus/MR was used to provide anodal tDCS to the dominant left M1. This was accomplished using the standard M1-SO electrode montage and two 5 × 7 cm rubber electrodes that were placed in sponges soaked in saline solution. Accordingly, the anode was placed over the left M1 location corresponding to the previously determined right FDI motor hotspot, whereas the cathode was placed over the contralateral (right) supraorbital region. The anode and cathode were held in these locations by a tightly fitting scalp cap. The typical rubber strap arrangement that normally is placed under the chin and over the anode was not used to hold the anode in place as this would have obviously interfered with the simultaneous TMS measurements. Fortunately, the tight scalp cap was able to hold the anode in position to the same degree as the normal rubber strap arrangement. However, the cathode was further secured using a rubber strap placed around the head in the typical fashion as this did not interfere with the TMS measurements. Overall, this arrangement was successful as although the TMS coil was relatively close to the tDCS electrodes, it did not touch them. tDCS was applied for a duration of 20 min at a current strength of 1 mA. The SHAM stimulation condition was administered in accordance with well-established procedures that have been shown to elicit the same sensations to participants as real tDCS, but without eliciting physiological effects [5,57]. Accordingly, the current was increased over a period of 10 s to 1 mA, kept constant at 1 mA for 30 s, and decreased down to zero over a period of 10 s as in previous studies [21,58]. Finally, the investigator who operated the tDCS device in the experiments did not participate in the data collection and the investigators who performed the data collection were blind to the experimental condition as described previously [21,45].

### 2.4. Data Analysis

MEP amplitude was the primary outcome measure, whereas RMT and the 1 mV SI were secondary outcome measures. All MEP and EMG data were collected using a custom Signal 5.04 software (Cambridge Electronic Design, Cambridge, UK) script and further analyzed offline using another custom Signal script. MEPs were quantified and expressed as the peak-to-peak amplitude and the mean of the 25 MEPs [56] in each TMS test block was used for analysis. MEP trials that had an average background EMG value greater than 25 microvolts in the 100 ms time window preceding the TMS artifact were discarded from the analysis. However, this only represented 5 total MEP trials out of the 4500 total MEP trials (0.11%) and had only a miniscule effect on the results.

### 2.5. Statistical Analysis

RMT and 1 mV SI were analyzed with two separate 2 *condition* (tDCS, SHAM) × 2 *hand* (Left, Right) within-subject ANOVAs. MEP amplitude was analyzed with a 2 *condition* (tDCS, SHAM) × 5 *test* (Pre, D5, D10, D15, Post) within-subject ANOVA. Post hoc analyses using Bonferroni adjustment for multiple comparisons were performed if appropriate to identify where significant differences occurred between the TMS test blocks. Finally, the effect sizes are given as the partial eta-squared values. The significance level was set to α < 0.05 for all statistical analyses. The data are presented as means ± standard errors in the figures and means ± standard deviation in the text. 

## 3. Results

### 3.1. RMT and 1 mV SI

**Figure 2:**
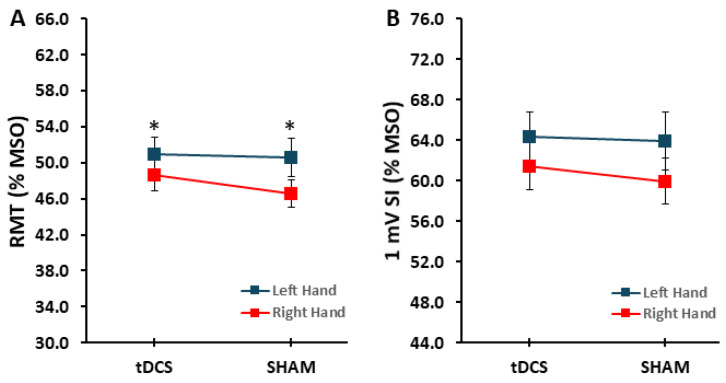
RMT and 1 mV SI. (**A**) RMT for the left and right hands in the tDCS and SHAM conditions. * indicates that the RMT was greater for the left hand compared with the right hand (*p* = 0.049); (**B**) 1 mV SI for the left and right hands in the tDCS and SHAM conditions.

The main effect for *condition* (F_[1,17]_ = 1.831, *p* = 0.194, η_p_^2^ = 0.097) and *condition* × *hand* interaction (F_[1,17]_ = 0.513, *p* = 0.483, η_p_^2^ = 0.019) were both non-statistically significant for RMT. However, there was a significant main effect for *hand* (F_[1,17]_ = 4.490, *p* = 0.049, η_p_^2^ = 0.209), which indicated that RMT was significantly lower in the FDI of the right hand compared to the FDI of the left hand (Figure 2A). For the 1 mV SI, the main effect for *condition* (F_[1,17]_ = 0.512, *p* = 0.484, η_p_^2^ = 0.029), the main effect for *hand* (F_[1,17]_ = 1.513, *p* = 0.235, η_p_^2^ = 0.082), and the *condition* × *hand* interaction (F_[1,17]_ = 0.192, *p* = 0.667, η_p_^2^ = 0.011) were all non-statistically significant (Figure 2B). 

### 3.2. MEP Amplitude

**Figure 3:**
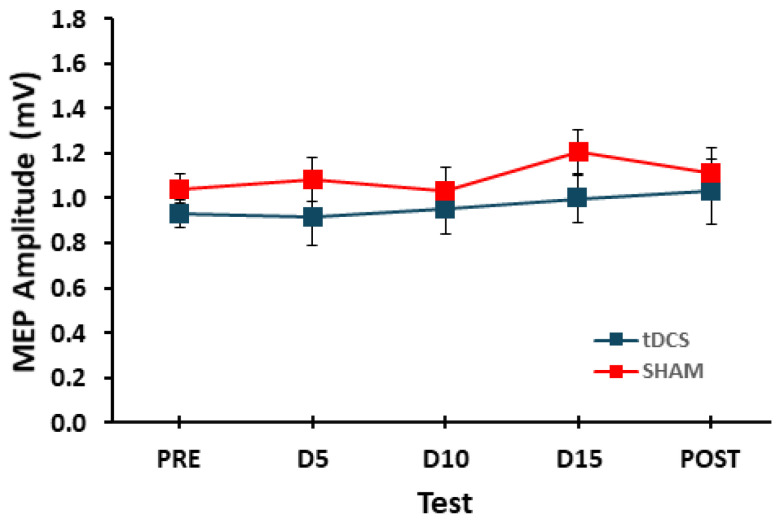
MEP amplitude for the tDCS and SHAM conditions in the Pre, D5, D10, D15, and Post TMS test blocks. Each data point represents the average of 25 MEPs recorded in each TMS test block.

For MEP amplitude, the main effect for *condition* (F_[1,17]_ = 1.629, *p* = 0.219, η_p_^2^ = 0.087), the main effect for *test* (F_[4,68]_ = 0.904, *p* = 0.467, η_p_^2^ = 0.050), and the *condition* × *test* interaction (F_[4,68]_ = 0.343, *p* = 0.848, η_p_^2^ = 0.020) were all non-statistically significant (Figure 3). 

## 4. Discussion

The purpose was to determine the influence of tDCS applied to the dominant left M1 on the excitability of the unstimulated contralateral non-dominant right M1. This study yielded two main findings. First, the MEP amplitudes evoked from the right M1 and collected from the corresponding FDI of the left hand were not statistically different between the tDCS and SHAM conditions at any timepoints during tDCS of the left M1. Second, the MEP amplitudes evoked from the right M1 were also similar between the tDCS and SHAM conditions immediately after the 20 min of stimulation of the left M1 ended. Taken together, the findings indicate that tDCS of left M1 does not modulate contralateral right M1 excitability during or immediately after application, at least under the current set of common tDCS parameters of stimulation. 

### 4.1. Influence of tDCS of Left M1 on Contralateral Right M1 Excitability

Numerous studies have reported that tDCS applied using the SO-M1 electrode montage for 10–20 min at 1–2 mA increases dominant left M1 excitability, as assessed by MEPs obtained from the contralateral dominant right hand that it primarily controls [7,8]. However, a non-trivial minority of studies have found no statistically significant effects of tDCS under those same experimental conditions [6]. To our knowledge, the present study was the first to examine the impact of this same set of tDCS parameters on the excitability of the unstimulated right non-dominant M1 by measuring MEP amplitudes obtained from the corresponding left non-dominant hand. Most importantly, a novel aspect of the present study was the focus on right M1 excitability during the exact time that tDCS was applied to left M1 as opposed to only undertaking post measurements. Due to obvious technical limitations, TMS measurements cannot be made from the stimulated M1 motor hotspot location during tDCS while the anode electrode is still in place. Accordingly, all tDCS studies that have measured the net excitability of the stimulated M1 have performed single-pulse TMS measurements before and after stimulation. In addition, in the one study available to our knowledge that investigated the effects of tDCS of the left M1 on the contralateral right M1 [25] also performed all TMS measurements before and after stimulation. Furthermore, the assessment of changes in IHI resulting from tDCS applied to M1 also cannot be accomplished during stimulation due to the same aforementioned TMS coil and anode electrode simultaneous spatial restrictions [26]. Overall, the effects of tDCS of the left M1 on the right M1 during tDCS could be functionally important as it is thought that applying tDCS during as opposed to before motor practice is more effective [1,23,24]. Furthermore, several lines of evidence indicate that the ipsilateral right M1 plays a significant role in both voluntary movement execution and motor learning of both hands [13,31,32,35,36,39].

The primary original hypothesis was that MEP amplitudes evoked from the right M1 would be greater both during and immediately after tDCS was applied to the left M1 compared to the SHAM condition. Although scant direct evidence existed and the available studies that were somewhat applicable were mixed [14,25], this hypothesis was predominantly based on an MRS study that found reduced GABA levels in both the unstimulated right M1 and the stimulated left M1 during and following tDCS [14]. These findings were also generally consistent with prior studies that had reported that reduced GABA levels (increased excitability) are one of the major neurochemical changes that occur in the stimulated M1 following application of anodal tDCS [3,59], although concentrations of other neuromodulators can also be modified [3]. Contrary to this original hypothesis, the MEP amplitudes evoked from the right M1 and collected from the corresponding left hand were not statistically different between the tDCS and SHAM conditions at any timepoints during or immediately after stimulation of the left M1. In addition, MEP amplitudes collected at all timepoints during and after tDCS application did not differ relative to the Pre TMS test block for either of the two stimulation conditions. Accordingly, MEP amplitudes in the TMS test blocks displayed no statistical changes over time and fluctuated about the average value (block grand average = 1.03 mV) for both stimulation conditions while displaying the well-known variability of MEP measurements (block standard deviation = 0.43 mV; range: 0.26–0.59 mV) [56].

These aforementioned results could not have been influenced by differences in control measurements such as baseline RMT and the 1 mV SI values employed for evoking MEPs. This is because these variables were nearly identical and not statistically different between the two stimulation conditions performed on the different days (Figure 2A,B). Although the common observation of a lower RMT for the right compared to the left hand was also found in the current study, the lack of a difference in RMT between stimulation conditions confirms that this hand difference had no effect on the overall outcomes. Most importantly, the methods used to obtain the 1 mV SI as a %MSO to elicit an average MEP of ~1 mV for the Pre TMS test blocks was successful. Accordingly, the Pre TMS test block had an average MEP amplitude very close to 1 mV for both the tDCS condition (0.93 ± 0.26 mV) and the SHAM condition (1.04 ± 0.27 mV) with no statistical difference between them. Therefore, there were no potentially confounding effects of a different SI as a %MSO or different MEP amplitudes evoked with this SI in the Pre TMS test blocks. In summary, the results from the control measures indicate that methodological factors or random variations in baseline cortical excitability between the two days could not have significantly influenced the overall MEP amplitude results obtained in all of the TMS test blocks. 

The current findings are seemingly not consistent with the primary study on which the original hypothesis was based [14]. This study was arguably the most relevant as it used MRS to measure GABA and glutamate concentrations in both M1s in response to tDCS of the left M1 using the SO-M1 montage. The major findings were that GABA levels were decreased compared to baseline in both the stimulated left M1 and the unstimulated right M1 during and following tDCS application, whereas glutamate levels were unchanged. Thus, direct physiological mechanisms were provided that could underlie the possibility that tDCS of the left M1 led to less inhibition and therefore greater excitation in both the left and right M1s. However, simultaneous measurements of MEPs were not recorded from either M1 in the study. In contrast, the present study did not include any other physiological measurements to complement the global net assessments of right M1 excitability as indicated by MEP amplitude. Therefore, it could not determine the possible modulation of any intercortical or intracortical pathways or neurochemical concentrations that may have contributed to the lack of MEP changes in right M1. It is possible that GABA concentrations were not changed in the right M1 in the current study, which could possibly reconcile the results of the two studies. Our findings are also potentially in conflict with the outcomes that would be predicted from current flow modeling studies of the electrical field produced by the SO-M1 electrode montage. For instance, a review article [60] illustrated (see their Figure 1A) that tDCS of left M1 leads to the generation of an electric field that, at least to some degree, impacts right M1 and several interconnected contralateral and ipsilateral brain areas that project to it. It is not unreasonable to assume that this should have led to some modulation of right M1 excitability whether up or down. Therefore, the absence of any modulation of MEP amplitudes in the present study implies that this pattern of current flow must have led to no net change in the balance of any inhibitory and excitatory right M1 circuits that it may have impinged upon. Finally, the findings are also generally incongruent with the bulk of the research literature that has reported significant enhancements in excitability following anodal tDCS in the M1 targeted by the stimulation [7,8], although a significant minority of studies have also reported no significant effects even in these circumstances [6].

In contrast, the current results are consistent with a prior study that had the most similar experimental design of any available study relative to the current one [25]. Although it appears that no existing studies have undertaken concurrent TMS measurements from the right M1 during tDCS of the left M1, this small-scale (*n* = 8) study [25] did perform analogous TMS measurements in the right M1 before and after anodal tDCS (1 mA) was delivered to the left M1 via the SO-M1 montage for 10 min. They reported that tDCS of the left M1 did not significantly change MEP amplitudes evoked from the right M1 and collected from the corresponding left hand immediately and 40 min after stimulation relative to the baseline. However, the study did not include a SHAM condition for comparison to the tDCS condition, although a cathodal tDCS condition also resulted in no changes in the MEP amplitudes evoked from the right M1. Nonetheless, the lack of change in MEPs collected from the left hand after anodal tDCS of the left M1 are congruent with the current findings. Taken together, the results of the two studies suggest that tDCS’s effects on cortical excitability are confined to the stimulated M1 both during and after stimulation. The findings are also in agreement with a study by Tazoe et al. (2014) [26] which found that anodal tDCS did not influence net contralateral M1 MEP amplitudes when measured after stimulation. However, paired-pulse TMS measurements indicated that it did increase the magnitude of IHI onto the contralateral M1. This finding is somewhat difficult to interpret based on the MEP results, but likely resulted from the influence of other pathways that acted in opposition to IHI, leading to no change in net global excitability as indicated by MEP amplitude. It should also be noted that the study involved the opposite experimental arrangement relative to the current study, as anodal tDCS was given to the right M1 with MEPs and IHI being measured in the left M1. This is important because the dominant M1 elicits greater IHI on the non-dominant hemisphere than vice versa [27]. Nevertheless, the outcomes remain generally consistent with the notion that the effects of anodal tDCS may be confined to the stimulated M1, at least when the SO-M1 electrode montage and a 1 mA current are used.

Indirect findings from the literature also support the current findings. Although the lack of changes in MEPs evoked from the right M1 were unexpected, it should be remembered that at least 20–30% of available tDCS studies that have investigated the excitability of the stimulated M1 have reported no significant effects [6,7,8]. Therefore, it would not be particularly surprising that it may be even more difficult to elicit significant excitability enhancements in an unstimulated distant region compared to a directly stimulated region, despite the strong anatomical interconnections between the two M1s. A demonstration of augmentations in right M1 excitability would have provided rather strong support for the idea that tDCS of the left M1 may not only improve right hand performance, but also the performance of the left hand. For example, a long-term motor learning study (no tDCS) involving only motor task practice of the right hand demonstrated that left hand motor skill was also substantially improved [36]. Crucially, this was accompanied by an increase in the excitability of the right M1 after several weeks of motor practice of the right hand. These two results and a number of other studies indicate that the ipsilateral (right) M1 plays an important role in voluntary movement control and motor learning of the right hand [13,33,34,35,36,37,38,39], despite it not being the hand it primarily controls. The current results indicate that tDCS of the left M1 does not increase right M1 excitability and therefore may not augment motor learning by impacting processes within the ipsilateral right M1. However, this interpretation is highly speculative as no motor skill training was carried out in the current study. Nonetheless, a few studies have investigated the influence of tDCS of the left M1 on the performance of the left hand that is primarily controlled by the right M1. These studies displayed extremely mixed results as two found a small non-significant increase in left hand motor performance [28,29], and one found a trend of a decrease [30]. Thus, these studies are in overall broad agreement with the current results as they collectively suggest that the effects of tDCS of the left M1 using the SO-M1 montage most likely neither increase or decrease left hand performance, but rather it likely has no discernable effects. 

### 4.2. Possible Mechanisms Underlying the Lack of tDCS of Left M1 Effects on Right M1

Theoretically, tDCS applied to the left M1 using the SO-M1 montage could impact the right M1 in at least three different general ways, although direct and systematic studies of these specific paths have not been performed. First, the most obvious and probable way would be as a result of the current flow from the cathode located over the right supraorbit to the anode located over the left M1 that typically leads to a net increase in left M1 excitability. Thus, the effects of this current flow on both intracortical neurons in left M1 and neuronal populations responsible for the amount of either IHI or IHF exerted on the right M1 are the most likely candidate mechanisms for any possible modulations of right M1 excitability. Second, this same flow of current could influence the right M1 due to possible effects it may have on the dorsolateral prefrontal cortex (DLPFC), supplementary motor complex (SMC), and especially the premotor cortex (PMC) ipsilateral to the left M1. For example, while left DLPFC does not have direct connections to either the right or left M1, paired-pulse TMS studies have shown short-latency inhibitory effects from left DLPFC onto the ipsilateral left M1 [61]. These are likely mediated indirectly through PMC, SMA, or the basal ganglia [61]. In contrast, the left dorsal PMC has dense connections not only to the ipsilateral left M1, but also to the contralateral right M1 [62,63,64]. Thus, right M1 excitability could potentially be elicited by the effects of current flow to the left DLPFC, SMC, and dorsal PMC and their collective ipsilateral connections to the left M1. Furthermore, contralateral connections to the right M1 could be involved that likely predominately emanate from left dorsal PMC [64]. Third, it is possible as indicated by current flow modeling studies [60] that anodal tDCS of the left M1 using the typical SO-M1 electrode montage generates a much more widespread modulation of activity in various brain regions than generally appreciated, even those beyond the previously discussed ipsilateral DLPFC, SMC, and PMC. This oftentimes underappreciated issue is described in a review [60] and illustrated (see their Figure 1) by the simulated electrical field distributions over the right hemisphere. Although the overall electrical field area and strength is lower for the right compared to the left hemisphere, it is shown that even a 1 mA current can clearly influence some of the more anterior portions of right M1 and to a greater extent areas located further anterior such as the corresponding ipsilateral right DLPFC, SMC, and PMC [60]. 

Despite these three general ways that right M1 excitability could be modulated by tDCS of the left M1 applied at a current strength of 1 mA using the SO-M1 montage, the complete absence of changes in MEP amplitudes evoked from the right M1 in the current study strongly suggests that none of these three individual paths and their underlying potential mechanisms of action exerted any meaningful influence on the right M1. In addition, the same would be true for any potential combination and relative weighting of these three paths on right M1 excitability. The current study did not include other physiological measures of right M1 activity. Thus, determination the exact physiological mechanisms for a lack of tDCS of left M1 effects on the right M1 was well beyond the scope of this study. Nonetheless, some potential factors responsible for the results can be briefly speculated upon based on the existing literature. 

It could be that tDCS simply does not elicit increases in excitability of the magnitude or consistency that many initial studies observed, even in the M1 directly targeted by anodal stimulation [6]. Support for this basic explanation comes from an older review article that concluded that the magnitude of tDCS effects [8] on M1 excitability declined with time as the methodology of these studies advanced. Accordingly, the most recent comprehensive review published on the topic, which uniquely focused on tDCS studies that induced no significant effects on M1 excitability, cited numerous studies that indicated no significant effects [6]. Specifically, the PRISM flow diagram of this study indicated that 92 studies were excluded due to finding significant results while 43 studies comprising a total of 47 experiments were included that reported no significant tDCS effects on M1 excitability. In particular, a recent comprehensive study with a rigorous design and a large sample size (*n* = 62) found no effects of anodal tDCS of the left M1 using the SO-M1 montage, although that study used a 2 mA current strength [22]. Another possible reason for the absence of significant effects in the current study is that the sample of participants could have had a relatively large number of tDCS non-responders [55,65,66]. While this is a definite possibility, Jonker et al. (2021) [22] also found no evidence for the existence of either responders or non-responders as indicated by a mixed-model cluster analysis. An additional explanation for the lack of significant findings that is typically cited in tDCS studies is possible differences in participant characteristics such as age, handedness, and gender distribution [6]. However, this explanation is likely not applicable to the current study as these characteristics were not substantially different in the study relative to previous studies [6,7,8]. Specifically, the current study involved all strongly right handed young adults in a tight age range with a gender distribution similar to the average of previous studies. Finally, the absence of effects was likely not due to the tDCS parameters utilized as the most common and efficacious parameters from the literature were employed. This is also apparent based on the data comparisons that a recent review [6] performed relative to prior reviews on the topic that focused on all available studies whether each study demonstrated significant tDCS effects on M1 excitability or not [7,8]. 

### 4.3. Limitations and Future Research Directions

Despite the relatively clear and straightforward findings, the present study had a number of limitations that should be acknowledged: (1) The 1 mA current strength may not have been high enough to induce significant changes in excitability in the unstimulated right M1 [25] to influence any potential mechanisms that could have led to enhanced right M1 excitability. On the other hand, several studies have shown that any increases in excitability of the stimulated M1 resulting from current strengths of 2 mA and above are non-linear [67,68]. Moreover, MEP amplitudes after 2 mA stimulation are often not significantly greater than current strengths of 1 mA and can even be lower [67]. Similarly, the study did not involve a condition involving cathodal tDCS of the left M1, which would have had to have been performed in an additional experimental session. While it seems doubtful that cathodal tDCS would have elicited an effect in the right M1 if anodal tDCS did not, this would need to be examined directly. Relatedly, this study did not investigate contralateral effects in the opposite direction (anodal tDCS applied to right M1), although once again it seems unlikely that statistically significant effects would have emerged. (2) The present study lacked longer-time-scale TMS testing blocks (e.g., 5–90 min) following the cessation of stimulation that are typically undertaken in tDCS studies of the targeted M1 [6,7,8]. While this would have been ideal, it seems highly unlikely that these test blocks would have demonstrated any differing relative results in light of the complete absences of MEP enhancement during and immediately after tDCS. (3) The net right M1 excitability as indicated by MEP amplitude elicited by single-pulse TMS was the only excitability metric acquired, and inclusions of paired-pulse TMS measurements such as short-interval intracortical inhibition (SICI) or intracortical facilitation (ICF) were not conducted [68]. These measurements were not undertaken because this study was focused on first determining overall net excitability changes in the right M1 if they were to exist. Additionally, SICI and ICF measurements were not performed due to the inherent time-related complications of having to elicit three types of responses for the requisite 25 trials for each variable (test MEP, condition–test MEPs for SICI and ICF, respectively) with a 6 s ITI. (4) Other more involved physiological measures involving techniques such as EEG or MRS were also not performed as they were not available and may not have even been technically feasible within the context of the current overall experimental paradigm. (5) A motor task was not utilized to concurrently quantify changes in motor skill of the left hand and their possible associations with any changes in right M1 excitability. This will have to be investigated in the future in more complex and longer-term experiments. (6) The sample size was a possible final limitation as this is an issue in many studies in different motor neuroscience related disciplines [69,70] as well as in most TMS and tDCS studies [6]. This was mitigated to a certain extent with the within-subject design and the moderately high sample size of 18, which was markedly higher than the average tDCS motor skill study (~13 per group) reported in the tables of an extensive review [1]. The sample size was also comparable (18 versus 19.7) to the average of sample size in 47 experiments included in a recent review of only non-statistically significant studies on tDCS effects on M1 excitability [6]. More importantly, the data provided in that paper indicated that the samples sizes (12.5 and 13.7) of previous reviews on the topic were even lower. This is important because both of those reviews concluded that tDCS significantly increased M1 excitability when all available significant and non-significant studies were included. Finally, the effect size (partial eta-squared) value obtained in the present study was very low (η_p_^2^ = 0.020) for the *condition* × *test* interaction for MEP amplitude, likely indicating that an unrealistically high number of participants would have been needed to achieve statistical significance. 

Future studies could investigate the interrelated issues of inter-individual differences in response to tDCS and the individualization of tDCS parameters based on the unique characteristics (e.g., anatomy, gender, genetics, hormonal profile, etc.) of a given participant. Furthermore, experimental designs should be more standardized across studies to better determine how a set of tDCS parameters that successfully enhance targeted M1 excitability and motor skill of the contralateral hand could also influence the unstimulated contralateral M1 and ipsilateral hand. These types of studies may be more valuable in older adult populations or in patients with motor disorders that exhibit degradations in hand dexterity or a differentially affected hemisphere (e.g., stroke, Parkinson’s disease) and corresponding hand. In addition, these studies would benefit from investigating multiple consecutive days of tDCS application in both healthy adults and patient populations. Another avenue of research would be to determine the association, if any, between changes in motor skill and changes in MEP amplitude in an analogous manner to that of studies involving the M1 targeted with tDCS. For instance, some early studies showed a positive correlation between changes in these variables [71,72]. Since classic work on motor learning (no tDCS involved) found MEP enhancements in task-specific muscles following complex fine motor skill acquisition [73,74,75], it was initially assumed that even further M1 excitability increases due to tDCS were partly responsible for motor skill improvements that were greater than practice alone. However, a series of more recent investigations [8,61,76,77,78] have shown that concurrent increases in motor skill and MEP amplitudes due to tDCS of the M1 are not significantly correlated. Therefore, it is highly possible that increased MEP amplitude values measured at rest may have limited functional relevance or at least not be directly causally related to acute motor skill enhancements. Nonetheless, all of these correlational studies had limitations and widely varying methodologies, which suggests that further data are needed before firm conclusions can be drawn on this issue, especially in regard to the unstimulated M1 and corresponding hand.

## 5. Conclusions

The current study represented a logical first step in the investigation of changes in right M1 excitability during and immediately after tDCS was delivered to the left M1. Therefore, this study was performed at rest to first understand the most basic effects of the stimulation without the complicating factors of simultaneous or previous general muscle contractions or specific motor practice. The main findings were that MEP amplitudes evoked from the right M1 and collected from the left hand were not statistically different between the tDCS and SHAM conditions at any timepoints during or after tDCS was delivered to the left M1. Furthermore, there were no significant increases in MEP amplitudes evoked from the right M1 in either stimulation condition compared to baseline. Collectively, the findings strongly suggest that tDCS does not modulate contralateral M1 excitability during or immediately after application, at least under the current set of common tDCS parameters of stimulation. However, substantially more future research is clearly needed to fully understand and characterize the influence of tDCS on the M1 contralateral to which it is applied. This will be challenging and require comprehensive studies that combine tDCS protocols with multiple physiological measures and a motor skill acquisition paradigm involving one or both hands.

## Data Availability

The data presented in this study are available on request from the corresponding author. The data are not publicly available due to privacy and ethical restrictions.
